# Ruminal Degradation of Puerarin and Its Effects on In Vitro Ruminal Fermentation, Methane Emission, and Microbial Community Structure

**DOI:** 10.3390/ani16010057

**Published:** 2025-12-24

**Authors:** Xiaomiao Guo, Zonglin Li, Xiaoqian Lin, Yushuang Pei, Zihui Wang, Yufei Ma, Yingmin Li, Hongjian Xu, Fengtao Ma, Yizhao Shen, Jianguo Li, Yanxia Gao

**Affiliations:** 1 College of Animal Science and Technology, Hebei Agricultural University, Baoding 071001, China; g1779480521@163.com (X.G.); 15530521751@163.com (Z.L.);; 2 Key Laboratory of Healthy Dairy Cattle Breeding of the Ministry of Agriculture and Rural Affairs (Co-Constructed by Ministry and Province), Baoding 071001, China; 3 Hebei Innovation Center of Cattle and Sheep Embryo Technology, Baoding 071001, China; 4 Hebei Research Institute of Dairy Industry Technology, Shijiazhuang 050221, China; 5 College of Animal Science and Technology, Shandong Agricultural University, Taian 271018, China

**Keywords:** puerarin, methane emission, rumen fermentation

## Abstract

Few studies have examined the effects of puerarin (PE) on ruminant parameters and methane production. Therefore, we determined the degradation of PE in the rumen and evaluated the effect of PE on in vitro fermentation, methanogenesis, and microbial community structure. A completely randomized design was used for the in vitro fermentation, and 4 gradient dosages of PE (0 mg/kg, 50 mg/kg, 100 mg/kg, and 150 mg/kg of DM) were applied in this trial. The in vitro fermentation was carried out in three runs at 6 h and 48 h, with four replicates per treatment per time point. Each run included 40 samples: eight treatments × four replicates and eight blank samples. Based on the fermentation results, both the PE treatments and the control group (CON) at 48 h were chosen for further analysis to explore the effects of PE on the bacterial community structure. Meanwhile, we determined the degradation rate and degradation products of PE in vitro ruminal fluid using high-performance liquid chromatography (HPLC). In this trial, PE may be isomerized into daidzin by rumen microorganisms; the in vitro degradation results of PE indicated that 70% of PE was degraded within 6 h, with the degradation rate reaching nearly 85% by 12 h. The concentrations of NH_3_-N and microbial crude protein (MCP) significantly increased linearly with the PE doses at 6 h (*p* = 0.01). The concentrations of MCP (*p* = 0.02) and propionate (*p* = 0.04) demonstrated a linear increase with increasing PE doses at 48 h. In contrast to microbial protein (MCP) and propionate, the acetate-to-propionate ratio decreased linearly with increasing PE doses at 48 h (*p* = 0.05). Additionally, the addition of PE linearly decreased methane production at 48 h (*p* = 0.01). Meanwhile, the relative abundances of *g_UBA1217* (*p* = 0.03), *g_UBA2810* (*p* = 0.04), and *g_Succiniclasticum* (*p* = 0.03) were significantly lower compared with the CON group. The results showed that PE can be degraded by rumen microflora. Furthermore, it can improve rumen fermentation parameters, increase the amount of protein synthesized by rumen microorganisms, and reduce methane production and the acetate-to-propionate ratio. PE could potentially be an effective strategy for methane mitigation; however, further research is needed to assess its in vivo effects in dairy cows over a longer period.

## Simple Summary

Currently, animal husbandry is a major contributor to global agricultural greenhouse gas emissions, with a substantial portion of its carbon footprint originating from methane produced by microbial fermentation in the rumen of ruminants. To mitigate this issue, one of the most effective strategies involves the application of natural feed additives. Puerarin (PE), a flavonoid compound with inherent bioactivities such as antibacterial properties, exhibits potential for reducing methane emissions. Therefore, this study aims to assess the effects of PE on nutrient digestibility and methane emissions, as well as its degradation dynamics in ruminal fluid. The results indicate that dietary inclusion of PE alters rumen fermentation and reduces methane emissions without affecting fiber degradability.

## 1. Introduction

Currently, the animal husbandry sector contributes significantly to global agricultural greenhouse gas emissions, with the majority of its carbon footprint stemming from methane produced via microbial fermentation in the rumen of ruminants [1]. In recent years, amid growing global climate concerns, substantial efforts have focused on developing methods to curb rumen methane production. Feed additives are widely recognized as effective, safe, and green agents with methane-inhibiting potential, thus garnering extensive research.

Puerarin, a flavonoid compound derived from kudzuvine root, is a component of traditional Chinese medicine. Numerous studies have shown that flavonoids possess a variety of biological activities [2,3]. In ruminants, flavonoids have been found to inhibit methane production and improve rumen fermentation [4]. Ma et al. [5] fed mulberry leaf flavonoids to sheep at a dose of 2 g/head per day, which effectively inhibited the abundance of methanogens without significant effects on rumen fermentation. Ehsan et al. [6] conducted in vitro experiments using seven flavonoids including rutin, quercetin, and catechin, and found that all of them significantly inhibited methane production except salicylic acid. Choi et al. [7] discovered that brown algae extract (rich in flavonoids) had a significant effect on the acetate/propionate ratio after in vitro fermentation, increasing propionate concentration while reducing methane production. Chusnul et al. [8] reported that although marigold flavonoids did not significantly inhibit methane production, they significantly increased the concentrations of propionate and microbial protein. In summary, flavonoids may improve rumen metabolic parameters, optimize rumen energy flow, and inhibit methane production. PE, similarly, is a type of flavonoid with carbonyl and polyhydroxyl structures. Based on this, we hypothesize that PE may exert similar effects to those reported in previous studies, potentially inhibiting methane production or improving rumen fermentation parameters.

Nevertheless, there appear to be no relevant studies that specifically investigate the effects of PE on dairy cows. Therefore, we aim to conduct a trial by adding PE to the total mixed ration (TMR) of dairy cows to observe its effects on milk yield and other parameters. Prior to the dairy cow trial, an in vitro fermentation trial should be conducted to assess its potential effects on fermentation parameters and ruminal microbial community structure. Thus, the objectives of this study were to explore the ruminal degradation of PE and its influence on in vitro ruminal fermentation, methane production, and microbial community structure.

## 2. Materials and Methods

This trial was conducted at the Hebei Agricultural University College (Baoding, China), and the animal procedures were approved by the Animal Care and Use Committee of Hebei Agricultural University College (GYX 1108).

### 2.1. Experimental Design

A completely randomized design was conducted for the in vitro fermentation, and 4 culture PE dose levels (0, 50, 100, and 150 mg/kg of DM) were used in this trial. The in vitro fermentation was carried out in 3 runs at 6 h and 48 h, and 4 replicates per treatment per time point. Each run included 40 samples: 8 treatments × 4 replicates and 8 blank samples. The added PE was completely soluble in pure distilled water, and the volume of distilled water added to the control group and the experimental group is the same. The standard PE (purity ≥ 98%) was procured from Beijing Suolaibao Technology Co., Ltd. (Beijing, China) Additionally, the dosage used in this trial is based on the Beijing Suolaibao Technology Co., Ltd. recommended dose.

### 2.2. In Vitro Incubation

Three ruminal cannulated lactating multiparous Holstein cows served as ruminal content donors (3 ± 1 of parity, 610.8 ± 50.3 kg of BW and 33.5 ± 2.0 kg of milk yield/d). These cannulated cows were fed a TMR consisting of corn silage (30%), alfalfa (20%), flaked corn (21.3%), soybean meal (5.6%), cottonseed meal (4.9%), beet granules (4.9%), bran (4.2%), dried distiller’s grains with solubles (7.2%), and minerals and vitamins (1.7%). Ruminal fluid was collected approximately 2 h before the morning feeding. Fluid from the three cannulated donor cows was mixed in equal volumes, transferred to a preheated, CO_2_-flushed insulated flask, and transported to the laboratory within 30 min. Subsequently, the ruminal fluid was filtered through four layers of gauze, with continuous CO_2_ gassing throughout the process to maintain anaerobic conditions. A ruminal dilution buffer was prepared according to the method described by Liu et al. [9]. The buffer solution was pre-warmed in a 39 °C water bath with continuous stirring and CO_2_ gassing, then mixed thoroughly with the filtered ruminal fluid at a 2:1 ratio (vol/vol) to prepare the inoculum. The fermentation system consisted of 40 bottles (120 mL volume), each containing an acetone-washed, pre-weighed ANKOM F57 fiber bag with (0.5050 ± 0.0010) g of feed substrate. The substrate was the same TMR fed to the donor cows, ground to pass through a 1 mm sieve. Each bottle was filled with 80 mL of the ruminal inoculum. The bottles were placed in an air bath incubator shaker (Jingda Instrument Manufacturing Co., Ltd., Jintan, Jiangsu, China) set at 39 °C with a shaking speed of 65 rpm for 48 h of fermentation. At the end of the incubation period, fermentation bottles were immediately placed in ice water to stop the fermentation. Samples were then collected and stored for subsequent analysis.

### 2.3. Sample Collection and Analysis

Cumulative gas was collected from each bottle at 6 h and 48 h using a pressure gauge (HCJYET, Hong Cheng Technology HT-935, Beijing, China) following the method described by Wang et al. [10]. A portable pH meter (Shanghai Yidian Science and Technology Co., Ltd., PHS-25, Shanghai, China) was used to measure the pH value of ruminal fluid samples. A liquid sub-sample was collected from each bottle: portions were stored in 10 mL tubes for the subsequent determination of VFA, NH_3_-N, and MCP; 2.5 mL aliquots were stored in tubes for 16S rDNA sequencing; additional aliquots were reserved for the determination of PE degradation rate; and separate portions were stored for the detection of PE metabolites. Specifically, samples for VFA and NH_3_-N analysis were stored at −20 °C, while those for MCP determination, determination of PE degradation rate, detection of PE metabolites, and 16S rDNA sequencing were stored at −80 °C. The concentration of VFA was measured using a gas chromatograph (Agilent Technologies, 7683B Series, Santa Clara, CA, USA) according to the procedures described by Hu et al. [11]. The NH_3_-N concentration was analyzed spectrophotometrically (UV-120-01, Shimadzu, Shanghai, China) following the method of Chaney and Marbach [12]. The content of microbial protein (MCP) was determined following the procedures outlined by Hu et al. [13]. The ANKOM F57 fiber bags were soaked and repeatedly rinsed with distilled water until the effluent was clear. They were then placed in a room-temperature incubator at 55 °C for 48 h, after which the disappearance rates of DM, NDF, and ADF were calculated. The contents of NDF and ADF were determined using a fiber analyzer (A200I, ANKOM, Macedon, NY, USA) according to the method described by Van Soest et al. [14]. The CH_4_ concentrations in individual airbags were quantified via an Agilent 7890 B gas chromatograph. This instrument was equipped with a packed Porapak Q column (Agilent Technologies) of dimensions 1 m × 2 mm × 3.175 mm, along with a thermal conductivity detector.

### 2.4. Determination of Puerarin Metabolism and Degradation

#### 2.4.1. Determination of Puerarin Metabolism

According to research by Zhong et al. [15], PE remains stable without decomposition at temperatures ranging from 30 to 90 °C. A total of 10 mL of ruminal fluid was collected at each fermentation termination time point (4, 6, 12, 24, and 48 h). The ruminal fluid was centrifuged at 12,000× *g* for 20 min, and the supernatant was transferred to an evaporating dish and dried at 65 °C. The dried residue was dissolved in 2 mL of methanol, sonicated for 10 min, and filtered through a 0.2 μm membrane filter. Subsequent analysis was performed using an ultra-high-performance liquid chromatography system (Laboratory of Beijing University of Chinese Medicine) equipped with a 2.1 mm× 100 mm, C18, 150 Å column (ACQUITY UPLC BEH). The mobile phase consisted of formic acid, water, and acetonitrile, with a flow rate of 0.4 mL/min and a detection wavelength of 150 nm. The peak area of PE in each sample was measured, and the concentration of PE was represented by its peak area. Non-linear regression analysis between PE concentration and degradation time was conducted using GraphPad Prism 9.0 to calculate the degradation rate and the R^2^ value of the model.

#### 2.4.2. Determination of Puerarin Degradation

In metabolomics analysis, 400 μL extract solution (Methanol: Acetonitrile = 1:1 containing isotopically labeled internal standard) was added to 100 μL ruminal fluid. Homogenization, sonication, and 40 °C incubation was first performed, the resulting mixture was then centrifuged (12,000 rpm, 15 min, 4 °C) to collect the supernatant. For LC-MS/MS analyses, a UHPLC system (Vanquish, Thermo Fisher Scientific, Sunnyvale, CA, USA) with a UPLC BEH Amide column (2.1 mm × 50 mm, 1.7 μm) coupled to an Orbitrap Expolris 120 mass spectrometer (Orbitarp MS, Thermo) was applied. After the raw data were converted to the mzXML format using Proteo Wizard software (V3.0.24054), metabolite identification was conducted with a collaboratively developed R package (3.3.5) and the BiotreeDB database (V3.0) was employed for this identification process [16]. The raw data included 3 quality control (QC) samples and 18 experimental. Subsequently, visual analysis was performed using an R package that has been fully developed by the Biotree research team, from which a total of 52,488 features were extracted. The raw data are available in the raw data table provided in the Results Section 3.

### 2.5. DNA Extraction and 16S rRNA Gene Sequencing and Analysis

Total genomic DNA samples were extracted using the MagBeads FastDNA Kit for Soil (116564384) (MP Biomedicals, Santa Clara, CA, USA) following the manufacturer’s instructions, and stored at −20 °C prior to further analysis. The quantity and quality of extracted DNA were measured using a NanoDrop NC2000 spectrophotometer (Thermo Fisher Scientific, Waltham, MA, USA) and agarose gel electrophoresis, respectively. Polymerase Chain Reaction (PCR) amplification of the bacterial 16S rRNA genes V3–V4 region was performed using the forward primer 338F (5′-ACTCCTACGGGAGGCAGCA-3′); (ACTCCTACGGGAGGCAGCA) and the reverse primer 806R (5′-GGACTACHVGGGTWTCTAAT-3′); (GGACTACHVGGGTWTCTAAT). Sample-specific 7 bp barcodes were incorporated into the primers for multiplex sequencing. Alpha-diversity metrics (Chao [17], Shannon [18], and Simpson [19]) and beta diversity metrics (weighted UniFrac, 2023.2 [20]) were estimated using the diversity plugin with samples rarefied to 50,944 sequences per sample. Taxonomy was assigned to ASVs using the classify-sklearn naïve Bayes taxonomy classifier in feature classifier plugin [21] against the SILVA Release 138 Database [22]. Principal component analysis (PCA) was also conducted based on the genus-level compositional profiles [23]. The taxonomy compositions and abundances were visualized using MEGAN (6.22.2) [24] and GraPhlAn (1.1.3) [25].

### 2.6. Statistical Analysis

Data processing in this trial was performed using SPSS 27.0 (SPSS Inc., Armonk, NY, USA). For all statistical analyses, the experimental unit was defined as the mean value of four replicate bottles within each run, with 3 independent runs performed. One-way ANOVA was applied to compare the significance of differences among groups, with in vitro fermentation parameters (pH, DMD, total gas, CH_4_, and VFA) analyzed using this method. Multiple comparisons were performed using the Tukey–Kramer test. Linear (L) and quadratic (Q) effects were used to evaluate the dose-dependent effects of PE on in vitro ruminal fermentation parameters, with each experimental unit (mean of four bottles per run) included in the regression analysis. The relative abundances of bacterial phyla and genera were compared using the Kruskal–Wallis H test with false discovery rate (FDR) correction, followed by the Scheffé test for multiple comparisons across treatment groups. A *p*-value < 0.05 was considered statistically significant, and values with 0.05 ≤ *p* < 0.10 were considered to indicate a tendency toward a difference.

## 3. Results

### 3.1. Determination of Puerarin Metabolism and Degradation

**Figure 1:**
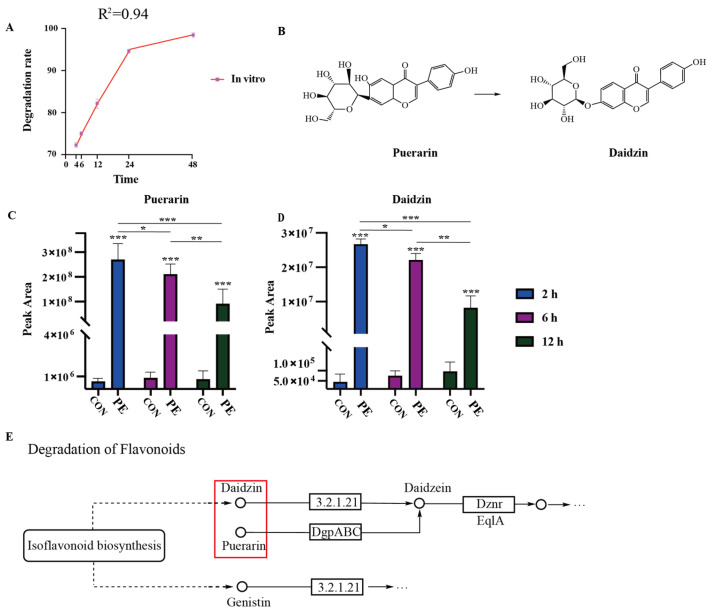
The degradation of puerarin in vitro with the increase in time (**A**). The chemical structural formulas of puerarin and daizin (**B**). The peak areas of puerarin and daidzin were detected by LC-MS/MS analyses (**C**,**D**). The partial degradation pathway diagram of puerarin from KEGG (**E**). * *p* < 0.05, ** *p* < 0.01, *** *p* < 0.001.

As shown in Figure 1A, the in vitro degradation results of PE indicate that 70% of PE was degraded within 6 h, and the degradation rate reached nearly 85% by 12 h. Figure 1B presents the chemical structures of PE and daidzin. Combined with the analysis of Figure 1C,D, it was found that PE may be isomerized into daidzin by rumen microorganisms. As shown in Figure 1C, the peak area of PE in the PE group was extremely significantly higher than CON (*** *p* < 0.001), and it decreased extremely significantly as time increased (*** *p* < 0.001). As depicted in Figure 1D, the peak area of daidzin in the PE group was also extremely significantly higher than CON (*** *p* < 0.001), and it showed an extremely significant decrease with the extension of time (*** *p* < 0.001).

### 3.2. The Effect of Puerarin on Fermentation Parameters of a TMR

In this trial, all data are presented in Table 1. No significant effect on gas production was observed (*p* = 0.35). A quadratic upward trend was noted as the concentration of the additive increased (*p* = 0.04). At 6 h, mean pH quadratically decreased (*p* = 0.01) and the amount of methane produced linearly decreased with increasing PE doses. However, there was no effect of treatment on DMD, NDFD, ADFD, and the concentration of VFA at 6 h. Additionally, the concentrations of NH_3_-N and MCP significantly linearly increased with the PE doses (*p* = 0.01).

The in vitro fermentation characteristics pH, NH_3_-N, MCP, VFA pattern, and CH_4_ are shown in Table 2. The pH values of each treatment group fluctuated between 6.54 and 6.58 with no significant difference. The NDFD (*p* = 0.13), ADFD (*p* = 0.35), and concentration of acetate (*p* = 0.55) remained stable, with no significant difference. Higher MCP concentration was observed in experiments compared with CON at 48 h (*p* = 0.04) and it increased quadratically with PE dose (*p* = 0.03). Total VFA concentration (*p* = 0.02) and gas production (*p* = 0.01) was significantly higher in both the 100 mg/kg and 150 mg/kg DM groups with CON, and it increased linearly with the PE dose (*p* = 0.01).

### 3.3. Changes in Microbial Community

**Figure 2:**
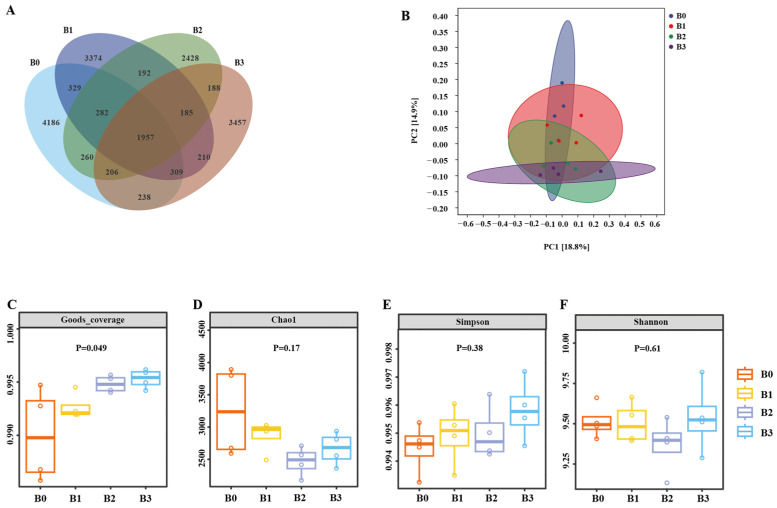
The effect of puerarin supplementation on microbial community. Veen graph (**A**), PCOA graph (**B**), Goods_coverage (**C**), Chao (**D**), Simpson (**E**), Shannon (**F**). B0 = Con, B1 = Con plus puerarin (50 mg/kg of DM), B2 = Con plus puerarin (100 mg/kg of DM), B3 = Con plus puerarin (150 mg/kg of DM).

As shown in Figure 2A, a total of 13,218 ASVs/OTUs were identified. Among these, 4186 ASVs/OTUs were unique to B0 (CON), 3374 were unique to B1 (50 mg/kg of DM), 2428 were unique to B2 (100 mg/kg of DM), and 3457 were unique to B3 (150 mg/kg of DM). As shown in Figure 2B, the *β*-diversity difference between the experimental group and the CON was significant (r = 0.2294, *p* = 0.0062). No statistically significant differences were observed in Chao (*p* = 0.17), Simpson (*p* = 0.38), and Shannon (*p* = 0.761) indices across treatments.

**Figure 3:**
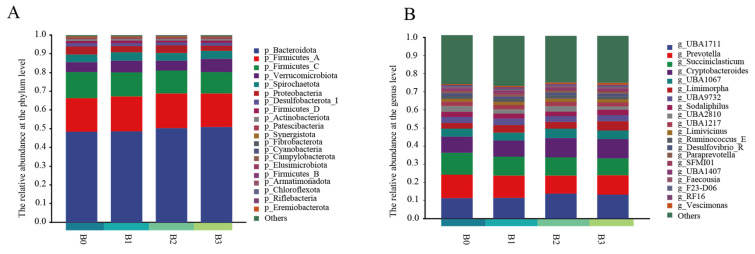
Phylum-level (**A**) and genus-level analyses (**B**) of puerarin addition after 48 h of in vitro fermentation. B0 = Con, B1 = Con plus puerarin (50 mg/kg of DM), B2 = Con plus puerarin (100 mg/kg of DM), B3 = Con plus puerarin (150 mg/kg of DM).

We employed the established method to perform phylum and genus analyses. A total of 20 phyla were identified. Five phyla were dominant, namely *Bacteroidota* (48.4%), *Firmicutes_A* (18.0%), *Firmicutes_C* (14.0%), *Proteobacteria* (4.3%), *Verrucomicrobiota* (5.1%), and *Spirochaetota* (4.2%) (Figure 3A). There were no significant differences between the experiment and CON at the phylum level (*p* = 0.38). At the genus level (Figure 3B), 20 genera were identified. Nine genera were dominant: *g_Prevotella* (12.6%), *g_Succiniclasticum* (12.0%), *g_UBA1711* (11.3%), *g_Cryptobacteroides* (8.8%), *g_UBA2810* (3.2%), *UBA1067* (3.5%), *Limimorpha* (4.3%), *Sodaliphilus* (2.5%), and *UBA9732* (3.3%). Among them, the relative abundance of *g_UBA 1217* (*p* = 0.03), *g_UBA 2810* (*p* = 0.04), and *g_Succiniclasticum* (*p* = 0.03) significantly decreased compared with CON (*p* = 0.03).

### 3.4. Analysis of Differences in Microbial Functional Pathways

**Figure 4:**
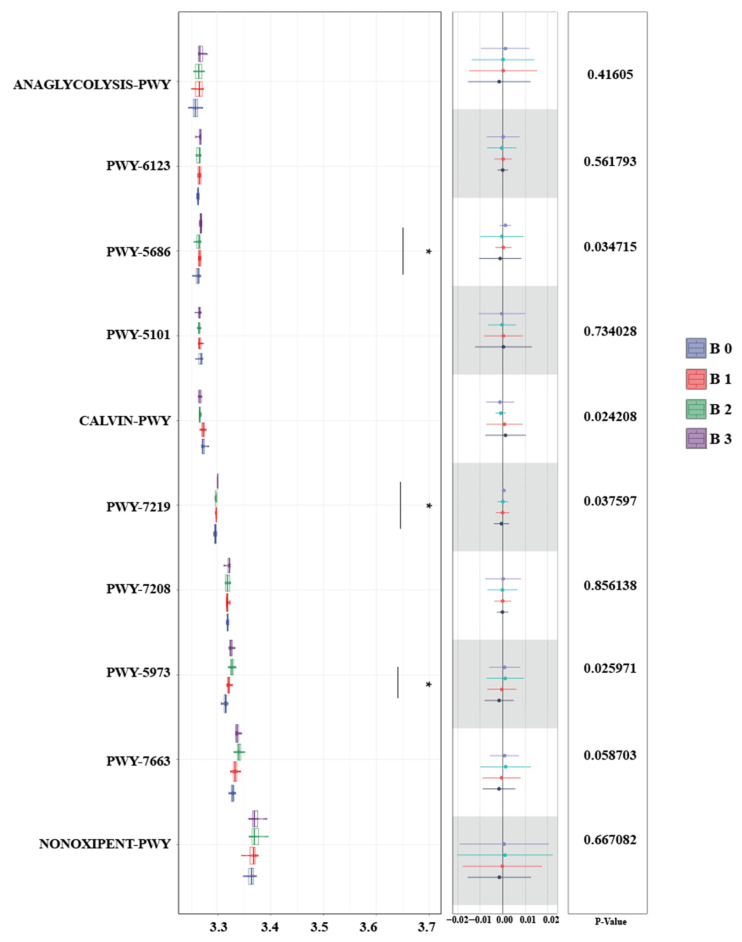
Functional differences in microbiota of puerarin after 48 h of in vitro fermentation with Kruskal–Wallis test. B0 = Con, B1 = Con plus puerarin (50 mg/kg of DM), B2 = Con plus puerarin (100 mg/kg of DM), B3 = Con plus puerarin (150 mg/kg of DM). * *p* < 0.05.

As shown in Figure 4, the Kruskal–Wallis test was used to analyze the differences in ruminal microbial functional pathways following PE supplementation. The results indicated that the pathways PWY-5686, PWY-7219, and PWY-5973 were significantly enriched compared with the CON.

## 4. Discussion

### 4.1. Determination of Puerarin Metabolism and Degradation

The degradation rate of PE in ruminal fluid has long been one of the key challenges in this field. Initially, we employed the sodium nitrite–aluminum nitrate–sodium hydroxide method for determination via ultraviolet spectrophotometry. However, the inherent color of the ruminal fluid changed over time, which significantly interfered with the accurate measurement by the spectrophotometer. Subsequently, we attempted to separate PE or a majority of it from the rumen fluid using polar organic solvents such as ethanol and ether to facilitate further analysis. Nevertheless, due to the unique properties of PE, efficient separation proved to be elusive. A review of the relevant literature revealed that PE maintains favorable properties even at 90 °C [15]. Therefore, we designed an experiment where rumen fluid containing PE was placed in an evaporating dish to evaporate the water, followed by dissolving the residue in methanol. The content of PE was then determined using ultra-high-performance liquid chromatography (UHPLC).

The metabolism of flavonoids by the rumen microbiota in ruminants primarily involves three pathways: hydrolysis, cleavage, and reduction [26,27]. Puerarin (PE) possesses an isoflavonoid chemical structure. Its B-ring, constrained by the steric hindrance of the carbonyl group in the pyran ring, forms an extended conjugated system, thereby adopting a nearly planar spatial configuration [28]. From the Kyoto Encyclopedia of Genes and Genomes (KEGG) pathway metabolic map, it is evident that both PE and daidzin can be hydrolyzed into daidzein, with glucuronic acid produced as a by-product [29,30,31,32]. In the present study, daidzin was detected at a significantly higher level in the PE group than in the CON l group at 2 h, suggesting that PE may be isomerized into daidzin by rumen microorganisms. This observation indicates the potential existence of a degradation pathway from PE to daidzin. However, the relative abundance of daidzin exhibited a time-dependent decrease, confirming that daidzin remains susceptible to microbial degradation. These findings imply that daidzin is likely an intermediate product of incomplete PE hydrolysis.

Since daidzin contains a glycosidic bond, it is theoretically feasible for microorganisms to hydrolyze it into glucuronic acid and daidzein [31]. Nevertheless, the present study failed to detect daidzein or observe a significant increase in the relative abundance of glucuronic acid. Additionally, the levels of 4′,5-dihydroxyflavone and 7,8-dihydroxyflavone (two isomers) in ruminal fluid increased inversely with the decreasing trend of daidzin, and the formation of these products cannot be explained based on chemical structure analysis. Thus, we hypothesize that PE may exhibit differences in both its degradation products and pathways between monogastric animals and ruminants, and the underlying mechanisms require further in-depth investigation.

### 4.2. Effects on In Vitro Fermentation Parameters

A study by Giller et al. [33] indicated that high-concentration polyphenolic compounds (plant extracts also contain multiple phenolic hydroxyl groups) can affect DMD. However, DMD was not negatively affected in this trial, which suggests that PE addition may not be bad for animal feed intake [9]. Another crucial pathway in ruminal fluid is the synthesis and absorption of nitrogen (N). After the addition of PE, both the concentrations of NH_3_-N and MCP increased linearly with increasing PE doses. This is a positive signal for the synthesis and absorption of ruminal protein, indicating an improved utilization rate of substrates by bacteria, which may be attributed to the enhanced protein degradation rate. The specific mechanisms may include the following two aspects: (1) The abundant phenolic hydroxyl groups in puerarin can form hydrogen bonds with amino acid residues (e.g., Gln153, Val162) and intercalate into protein molecules, leading to a reduction in the α-helix and β-sheet structures of proteins and thus disrupting their native conformations. (2) The phenolic hydroxyl groups in PE are easily oxidized to quinones, which undergo nucleophilic addition reactions with sulfhydryl groups. This reduces the protective effect of disulfide bonds, results in a decrease in the number of disulfide bonds, makes proteins more susceptible to the action of digestive enzymes, and thereby improves their water solubility and digestibility [34]. Initially, we hypothesized that plant extracts might inhibit the fermentation of feed by ruminal microorganisms due to their antibacterial properties [35], but subsequent parameter determinations negated this hypothesis. The nutrient degradation rate of the treatment was not impaired; on the contrary, some parameters were significantly higher than those of the CON (*p* < 0.05). This suggests that plant extracts may alter the ruminal fermentation pattern, directing more energy toward nutrient production rather than losing it in the form of gases (e.g., methane, CH_4_). In a study by Wang et al. [36], [H] (hydrogen protons)—the substrate for methane—were competitively utilized by propylene glycol to generate propionate, and the production of propionate showed a strong negative correlation with methane production. Battelli et al. [37] found that quercetin could inhibit methane production, while Chen et al. [38] reported that mulberry leaf flavonoids had no significant methane-inhibiting effect. In this experiment, a linear decreasing trend in methane production was also observed with the increase in PE addition. Meanwhile, a significant trend toward increased propionate content was noted, which indicates that [H] was diverted to the production of VFAs such as acetate and propionate, thereby altering the fermentation pattern [4]. Furthermore, the relevant literature points out that flavonoids can inhibit methanogenic bacteria (e.g., ciliates) [4,38,39,40]. Therefore, we speculate that PE may have the effect of inhibiting methane production.

### 4.3. Effects on Microbial Community

In ruminants, a symbiotic relationship exists between ruminal microorganisms and the rumen itself—a dynamic interplay that underpins the normal functioning of the rumen ecosystem [41]. The rumen microbial community serves as the core driver, orchestrating key metabolic processes such as ruminal fermentation, methane production, and biosynthesis of essential substances. Notably, in this trial, PE treatment exerted no significant impact on the Chao, Simpson, and Shannon indices of the ruminal microbial community, indicating that PE did not alter the richness, evenness, or overall diversity of the rumen microbial population. Meanwhile, the results showed that at the phylum level, the addition of PE had no significant effect on microorganisms. Firmicutes and Bacteroidetes were the dominant phyla, which was consistent with the findings of previous studies [42,43]. In this trial, the abundance of the genus *g_UBA1711* has an upward trend compared to CON. Kim et al. [44] analyzed adult buffaloes and found a significant positive correlation between the abundance of the genus *g_UBA1711* and lignin degradation. This is consistent with the results of this experiment [45]—in this trial, after the addition of PE, the ADFD showed an upward trend.

A growing body of research has established the existence of “hydrogen competition” between methane and propionate biosynthesis in the rumen, and congruent findings were obtained in the present study [43,46]. Specifically, the concentrate of methane and propionate exhibited an inverse correlation, with methane levels declining as propionate levels increased. This observed inverse relationship may be associated with a reduction in the relative abundance of *g_Succiniclasticum* [47]. Notably, *g_Succiniclasticum* is classified as a succinic acid-utilizing bacterium, as it possesses the metabolic capability to synthesize propionate via succinic acid metabolism. However, in this trial, *g_Succiniclasticum* displayed a reduction in response to PE exposure. Concomitantly, a reduction in methane production was observed, and this trend was consistent with the diminished abundance of *g_Succiniclasticum*—a result that corroborates the earlier findings reported by Bharanidharan et al. [48]. Furthermore, additional observations from this study revealed that the bacterial genera *g_UBA1217* and *g_UBA2810* exhibited sensitivity to PE, suggesting potential regulatory effects of this compound on these rumen microbial taxa.

### 4.4. Microbial Functional Pathways

Microbial metabolic pathways play a vital role in regulating rumen function [49]. In this trial, we investigated the potential impacts on the energy flow of ruminal microbes by predicting the enrichment of rumen microbial metabolic pathways following PE supplementation. From the viewpoint of microbial functional pathways, PWY-5686, PWY-7219, and PWY-5973 were significantly enriched in comparison to the control group. Among these, PWY-5686 is associated with UMP biosynthesis I. Within this pathway, Uridine 5′-monophosphate (UMP) and Cytidine Triphosphate (CTP) are ultimately synthesized from L-glutamine and bicarbonate. As downstream products of this pathway, both UMP and CTP are nucleotide compounds, which, similar to ATP in living organisms, can release the energy from high-energy phosphate bonds for energy transfer. Meanwhile, the PWY-7219 pathway is linked to ATP synthesis. An increase in ATP synthesis implies that more energy becomes available for rumen microbes. In this trial, the enrichment of the PWY-5686 and PWY-7219 pathways suggests that PE may influence energy flow by affecting the microbial UMP and ATP synthesis pathways. This also indirectly corroborates the increase in volatile acid and N content observed in the fermentation parameters. Additionally, the PWY-5973 pathway is the palmitoleic acid biosynthesis pathway, through which microbes produce cis-vaccenate that possesses a variety of biological functions. Overall, by predicting the influence of PE on microbial metabolic pathways, it is revealed that PE has potential functions in regulating microbial energy flow.

## 5. Conclusions

In conclusion, puerarin is susceptible to degradation by rumen microorganisms. When supplemented at a dosage of 150 mg/kg DM, PE exerted a significant inhibitory effect on ruminal methane emissions, reducing methane production by approximately 13.17%. Furthermore, PE induced distinct shifts in the rumen fermentation pattern, characterized by a notable reduction in the acetate-to-propionate ratio. Concomitantly, it enhanced ruminal nitrogen cycling efficiency and led to a marked increase in microbial protein synthesis. Based on these comprehensive findings, an optimal PE feeding rate in this in vitro study appeared to be 150 mg/kg DM for dairy cows. However, further research is needed to verify the in vivo effect of PE over a longer period.

## Figures and Tables

**Table 1:** Effects of the PE on CH_4_ production, VFA profiles, and other fermentation parameters in vitro at 6 h (PE; *n* = 12).

	Puerarin Dose ^2^ (mg/kg of DM)				SEM	*p*-Value ^3^	
Item ^1^	Con	50	100	150		L	Q
pH	6.76	6.78	6.78	6.77	0.003	0.22	0.03
DMD, %	28.19	28.63	29.29	29.63	0.012	0.01	0.05
NDFD, %	12.00	12.36	12.54	12.32	0.014	0.34	0.09
ADFD, %	9.53	9.24	9.56	9.83	0.019	0.47	0.41
NH_3_-N mg/dL	7.19 ^b^	7.99 ^b^	8.16 ^a^	8.37 ^a^	0.179	0.05	0.04
MCP mg/dL	27.55 ^c^	31.98 ^b^	32.19 ^a^	36.04 ^a^	0.865	0.04	0.35
Concentration (mM)							
Total VFA	20.91	22.36	22.51	22.74	0.370	0.07	0.58
Acetate	12.13	12.31	12.24	12.17	0.240	0.76	0.53
Propionate	5.42	6.97	6.99	7.15	0.110	0.73	0.37
Isobutyrate	0.13	0.12	0.12	0.13	0.003	0.74	0.12
Butyrate	2.38	2.25	2.42	2.49	0.483	0.27	0.32
Isovalerate	0.33	0.30	0.32	0.33	0.006	0.58	0.11
Valerate	0.48	0.43	0.46	0.47	0.009	0.87	0.25
A/P	2.07	2.06	2.04	2.04	0.009	0.61	0.67
Total gas production (mL)	54.21	52.20	54.15	57.66	0.770	0.03	0.01
CH_4_ (mL)	9.12 ^a^	8.83 ^ab^	8.62 ^b^	8.54 ^b^	3.151	0.01	0.01

^1^ DMD = apparent disappearance of dry matter (DM); NDFD = apparent disappearance of NDF; ADFD = apparent disappearance of ADF; MCP = microbial crude protein. ^2^ Data were analyzed using PE doses of 0, 50, 100, and 150 mg/kg of DM. ^3^ L = linear; Q = quadratic. ^a–c^ Means within a row with different superscripts differ (*p* < 0.05).

**Table 2:** Effects of PE on CH_4_ production, VFA profiles, and other fermentation parameters in vitro at 48 h (PE; *n* = 12).

	Puerarin Dose ^2^ (mg/kg of DM)				SEM	*p*-Value ^3^	
Item ^1^	Con	50	100	150		L	Q
pH	6.54	6.58	6.58	6.57	0.008	0.28	0.12
DMD, %	45.69 ^b^	46.23 ^a^	46.65 ^a^	47.61 ^a^	0.021	0.02	0.06
NDFD, %	34.31	34.60	34.34	35.17	0.015	0.54	0.26
ADFD, %	28.47	28.66	27.91	29.36	0.014	0.74	0.27
NH_3_-N mg/dL	13.67	13.42	13.18	13.64	0.189	0.45	0.54
MCP mg/dL	35.34 ^b^	37.77 ^a^	40.14 ^a^	41.01 ^a^	0.491	0.02	0.03
Concentration (mM)							
Total VFA	35.42 ^b^	35.78 ^b^	37.46 ^a^	37.57 ^a^	0.571	0.01	0.26
Acetate	18.99	19.12	20.14	20.49	0.278	0.25	0.50
Propionate	9.68 ^b^	9.69 ^ab^	10.70 ^a^	10.81 ^a^	0.166	0.04	0.06
Isobutyrate	0.29 ^b^	0.30 ^ab^	0.33 ^a^	0.32 ^a^	0.007	0.04	0.11
Butyrate	4.81 ^b^	4.98 ^b^	4.41 ^a^	4.14 ^a^	0.092	0.05	0.11
Isovalerate	0.80 ^b^	0.83 ^b^	0.84 ^a^	0.88 ^a^	0.022	0.01	0.01
Valerate	0.85 ^b^	0.86 ^b^	1.01 ^a^	0.93 ^a^	0.024	0.05	0.10
A/P	1.96 ^a^	1.93 ^ab^	1.88 ^b^	1.89 ^b^	0.013	0.05	0.07
Total gas production (mL)	96.78 ^b^	97.65 ^b^	99.42 ^a^	101.25 ^a^	2.861	0.01	0.01
CH_4_ (mL)	12.68 ^a^	11.93 ^ab^	11.44 ^b^	11.01 ^b^	2.112	0.01	0.01

^1^ DMD = apparent disappearance of dry matter (DM); NDFD = apparent disappearance of NDF; ADFD = apparent disappearance of ADF; MCP = microbial crude protein. ^2^ Data was analyzed using PE doses of 0, 50, 100, and 150 mg/kg of DM. ^3^ L = linear; Q = quadratic. ^a–b^ Means within a row with different superscripts differ (*p* < 0.05).

## Data Availability

The original contributions presented in this study are included in the article. Further inquiries can be directed to the corresponding author.
